# Deaths in England under Anæsthetics

**Published:** 1901-03

**Authors:** 


### DEATHS IN ENGLAND UNDER ANÆSTHETICS.

The number of deaths reported recently in the English journals during the administration of anæsthetics, and especially nitrous oxide, has created attention not unmingled with astonishment upon this side of the water.

The rarity of this in America, either in medical or dental practice, leads to the conclusion that there must be something wrong in the training of those who assume to give anæsthetics.

The latest, which is given upon another page under the heading, "Death under Nitrous Oxide," might better have been called criminal negligence. The fact that the patient died from a sponge used to absorb blood and allowed to remain in the mouth and drawn in "and firmly impacted in the air-passages" is almost beyond credence.

It was certainly kind in the coroner to express sympathy with the operator, but the jury's verdict of "death by misadventure" rather exceeds anything in the form of coroners' verdicts that has been noticed in the literature of coroners' inquests.

That accidents may happen in the administration of anæsthetics is too well known to need comment, but the case in question does not seem to belong to this category.

It is true that daily and hourly association with agents and appliances, more or less dangerous, causes a feeling of indifference, and the wonder is that accidents of a serious nature are not more common in practice. It is probable that few take any special precautions in the use of clamps, yet the possibility of the clamp breaking under undue strain or molecular change through constant use seems to be unnoticed. In the writer's experience this has occurred several times, and but for precautionary measures might have resulted seriously to the patient.

Teeth slipping through the beaks of the forceps have caused serious results. This can scarcely be claimed as one of the avoidable accidents, but it should be guarded against by careful examination of the beaks of the forceps that they are not worn smooth by constant use.

Carelessness in the use of escharotics has resulted in serious lesions, which, while not endangering life, have not infrequently produced sloughing and painful injuries to patients.

The number of these results of careless operations have multiplied in proportion to the increase of powerful therapeutic agents and appliances to be used in the oral cavity, and the question must be seriously asked, Are we taking all possible precautions to prevent injury to those coming under our care?

